# Helminths are positively associated with atopy and wheeze in Ugandan fishing communities: results from a cross‐sectional survey

**DOI:** 10.1111/all.12867

**Published:** 2016-05-20

**Authors:** E. L. Webb, M. Nampijja, J. Kaweesa, R. Kizindo, M. Namutebi, E. Nakazibwe, G. Oduru, P. Kabubi, J. Kabagenyi, G. Nkurunungi, D. Kizito, L. Muhangi, M. Akello, J. J. Verweij, B. Nerima, E. Tukahebwa, A. M. Elliott, the LaVIISWA trial team, Richard Sanya, Beatrice Mirembe, Justin Okello, Jonathan Levin, Christopher Zziwa, Josephine Tumusiime, Moses Sewankambo, Denis Nsubuga, Stephen Cose, Linda Wammes, Emmanuel Niwagaba, Grace Kabami, Elson Abayo, Fred Muwonge, David Abiriga, Victoria Nannozi, James Kaweesa

**Affiliations:** ^1^ London School of Hygiene and Tropical MedicineLondonUK; ^2^ MRC/UVRI Uganda Research Unit on AIDSEntebbeUganda; ^3^ Vector Control DivisionMinistry of HealthKampalaUganda; ^4^ Entebbe HospitalEntebbeUganda; ^5^ Laboratory for Medical Microbiology and ImmunologySt Elisabeth HospitalLC Tilburgthe Netherlands

**Keywords:** allergy, atopy, helminths, household survey, wheeze

## Abstract

**Background:**

Parasitic helminths are potent immunomodulators and chronic infections may protect against allergy‐related disease and atopy. We conducted a cross‐sectional survey to test the hypothesis that in heavily helminth‐exposed fishing villages on Lake Victoria, Uganda, helminth infections would be inversely associated with allergy‐related conditions.

**Methods:**

A household survey was conducted as baseline to an anthelminthic intervention trial. Outcomes were reported wheeze in last year, atopy assessed both by skin prick test (SPT) and by the measurement of allergen‐specific IgE to dust mites and cockroach in plasma. Helminth infections were ascertained by stool, urine and haemoparasitology. Associations were examined using multivariable regression.

**Results:**

Two thousand three hundred and sixteen individuals were surveyed. Prevalence of reported wheeze was 2% in under‐fives and 5% in participants ≥5 years; 19% had a positive SPT; median *Dermatophagoides*‐specific IgE and cockroach‐specific IgE were 1440 and 220 ng/ml, respectively. *S. mansoni, N. americanus*,* S. stercoralis*,* T. trichiura, M. perstans* and *A. lumbricoides* prevalence was estimated as 51%, 22%, 12%, 10%, 2% and 1%, respectively*. S. mansoni* was positively associated with *Dermatophagoides*‐specific IgE [adjusted geometric mean ratio (aGMR) (95% confidence interval) 1.64 (1.23, 2.18)]; *T. trichiura* with SPT [adjusted odds ratio (aOR) 2.08 (1.38, 3.15)]; *M. perstans* with cockroach‐specific IgE [aGMR 2.37 (1.39, 4.06)], *A. lumbricoides* with wheeze in participants ≥5 years [aOR 6.36 (1.10, 36.63)] and with *Dermatophagoides*‐specific IgE [aGMR 2.34 (1.11, 4.95)]. No inverse associations were observed.

**Conclusions:**

Contrary to our hypothesis, we found little evidence of an inverse relationship between helminths and allergy‐related outcomes, but strong evidence that individuals with certain helminths were more prone to atopy in this setting.

Allergy‐related diseases increased dramatically in affluent and middle‐income countries during the twentieth century 1, 2: asthma now affects about 300 million people 3, and eczema 5–20% of children 4. Although these conditions remain relatively rare in low‐income countries (LICs), they appear to be increasing 5, 6. It has been hypothesized that helminth infections, still highly prevalent in LICs 7, may contribute to these geographic differences. Globally, the majority of asthma and eczema cases are associated with allergen sensitization or atopy. However, in LICs, the role of atopy in allergy‐related disease is less certain 8, 9.

Parasitic helminths evolved to coexist with their mammalian hosts, are often asymptomatic and cause limited mortality. To this end, they have developed mechanisms for evading or modulating the host immune response. Evidence from animal models, and from *in vitro* studies on human samples, suggests that helminths can modulate the immune response not only to themselves, but also to unrelated pathogens, antigens and allergens 10, 11. Helminths contain a range of molecules homologous to known allergens, but absent from mammals or markedly different from mammalian homologues. These induce IgE responses in mammalian hosts, and there is a strong evidence that this pathway is involved in protective immunity against helminths 12. Modulation of this atopic pathway is likely, therefore, to be particularly important for helminth survival, while concomitantly protecting against allergic disease 13.

Associations between helminths and allergy‐related conditions have been investigated in a variety of settings [reviewed in 14, 15, 16]. The majority of studies investigating helminth–atopy associations have either reported an inverse association or no evidence of association. However, results for allergy‐related diseases such as asthma and eczema have been less consistent, varying both within and across helminth species. Meta‐analyses have indicated that for asthma, *Ascaris lumbricoides* infection is positively associated and hookworm inversely associated, with borderline evidence of a positive association for *Trichuris trichiura* and no association for *Strongyloides stercoralis* 15. Very few studies have investigated associations with schistosomiasis, although those that have generally report an inverse association with atopy 17, 18, 19, 20.

Helminths may have an important role as modifiers of associations between markers of atopy and allergy‐related diseases. It has been reported that IgE and SPT responses to house dust mite are positively associated in the absence of hookworm but unrelated among hookworm‐infected individuals 21. Similarly, studies have found that positive associations between atopy and wheeze, and between atopy and eczema, exist only among individuals who do not have hookworm and not among those infected with hookworm 22, 23. Finally, it has been reported that atopy and wheeze are only positively associated in the absence of concurrent *Ascaris* infection and not in its presence 24.

Intervention studies are an important tool for understanding the relationship between helminths and allergy‐related diseases; however, findings of previous studies on the impact of anthelminthic treatment on atopy among children are conflicting 25, 26, 27, 28 and none showed effects on wheeze or eczema, although statistical power for these outcomes was usually limited. Variable findings for atopy may be a consequence of heterogeneity between study settings in helminth species; of note, no trial has yet investigated the effects of treatment of schistosomiasis on asthma, eczema and atopy.

We designed a cluster‐randomized trial, the Lake Victoria Island Intervention Study on Worms and Allergy‐related Diseases (LaVIISWA; ISRCTN47196031), to investigate the impact of intensive *vs* standard anthelminthic treatment over a 3‐year period on allergy‐related diseases, in a setting with heavy helminth burden, in particular schistosomiasis 29. We herein report the findings of a household survey, conducted as baseline, to investigate the hypothesis that helminth infections would be inversely associated with allergy‐related conditions.

## Methods

### Study design and procedures

LaVIISWA is being conducted in 26 fishing villages on the Lake Victoria islands of Koome subcounty, Mukono district, Uganda, a remote setting accessible in 2–3 h from Entebbe by powered canoe. Full details of the trial design are described elsewhere 29. The baseline household survey was conducted between October 2012 and July 2013, across all trial villages, immediately preceding intervention roll‐out. All households in participating villages were eligible for inclusion in the survey. Available household listings were checked and updated by the research team, and simple random samples of 45 households were selected from each village. In selected households, all members were eligible for inclusion in the survey.

Questionnaires were completed regarding household features and individual social‐demographic characteristics. Information regarding asthma, eczema and allergy symptoms was obtained using questions from the International Study on Allergy and Asthma in Children (ISAAC) questionnaire, with supplementary questions from the UK diagnostic criteria for atopic eczema 30, 31.

A general history and examination, including height, weight and hepatosplenomegaly, was performed. All individuals were examined for visible flexural dermatitis: for this, all team members were trained in the standardized approach described in 32. SPTs were performed on participants aged ≥1 year, using standard methods, with three allergens [*Dermatophagoides* mix, *Blomia tropicalis* and German cockroach (*Blatella germanica*)] and positive and negative controls (ALK‐Abelló; supplied by Laboratory Specialities (Pty) Ltd., Randburg, South Africa). Each participant was asked for one stool sample; mid‐stream urine samples were requested from all participants in the 15 villages surveyed from February 2013 onwards. Blood samples of 14 ml were obtained from individuals ≥13 years, 10 ml from children 5–12 years and 6 ml from children 1–4 years. Individuals were offered HIV counselling and testing in collaboration with local health service providers.

Ethical approval was granted by the Research and Ethics Committee of the Uganda Virus Research Institute, the Uganda National Council for Science and Technology, and the London School of Hygiene and Tropical Medicine. Individual written informed consent (for adults ≥18 years and emancipated minors, and for children by a parent or guardian) and assent (for children 8–17 years) was sought for survey participation.

### Laboratory methods

Two slides from each stool sample were examined (by different technicians) using the Kato‐Katz method 33. The remaining sample was suspended in ethanol and stored at −80°C to allow further investigation for *Necator americanus* and *Strongyloides stercoralis*, and, among a subset of 200 participants, for *Ancylostoma duodenale*, using real‐time polymerase chain reaction (RT‐PCR) 34. Quality control for PCR assays was conducted at St Elisabeth's Hospital, Tilburg, NL. The Uganda results were comparable for *N. americanus* and *A. duodenale*, but had a lower detection rate for *S. stercoralis*. The presence of circulating cathodic antigen (CCA) of *S. mansoni* in urine was assessed (Rapid Medical Diagnostics, Pretoria, South Africa). Infection intensity based on Kato‐Katz results was classified using WHO‐recommended cut‐offs 35. For PCR results, there are no standard cut‐offs for categorizing infection intensity; however, based on results from Verweij et al. 34, individuals with *C*_t_ > 30 would have parasite loads difficult to detect by microscope. *Mansonella perstans* infection was determined by a modified Knott's method 36; malaria was determined by thick blood film.

IgE specific to *Dermatophagoides* and cockroach allergens was measured by ELISA 29. The lower detection limit for our in‐house ELISA was 15.6 ng/ml. We used 20‐fold diluted plasma samples in our assay; hence, the lower detection limit in undiluted plasma was calculated as 312 ng/ml. This was used as a cut‐off to create binary variables for detectable *vs* undetectable responses for each allergen.

### Statistical methods

This was a cross‐sectional analysis of survey data. Outcomes were reported wheeze in the last 12 months for children <5 years and for participants ≥5 years; visible flexural dermatitis; atopy defined as positive SPT response to any allergen for participants ≥1 year; atopy assessed as concentration of asIgE and analysed both as a continuous outcome and as detectable/nondetectable using the cut‐off of 312 ng/ml. Exposures for the analysis were helminth infections. The following variables were considered as potential confounders: individual socio‐demographic characteristics (age, sex, birth order, number of siblings, area of birth, area resided in for first 5 years, preschool attendance; occupation, maternal tribe, paternal tribe); behavioural and clinical characteristics (hand‐washing behaviour, BCG scar, maternal or paternal allergy/asthma/eczema, immunization history, breastfeeding, exposure to anthelminthic treatment *in utero*, anthelminthic treatment in last 12 months, artemisinin combination treatment for malaria in last 12 months, malaria infection, HIV infection); and household characteristics (crowding, animal ownership, asset score, indoor cooking, toilet access, drinking water source, washing water source, malaria control measures).

Assuming an average of 2.8 people per household, we expected that sampling 45 households per village would yield at least 3250 participants. For common exposures (prevalence ≥20%), assuming a design effect of 1.5 and outcome prevalence of 10%, the study would have over 80% power to detect risk ratios ≥1.5.

All analyses employed the ‘svy’ survey commands in Stata to allow for clustering of respondents within villages using linearized standard errors 37 and for variable village sizes using weights. Village‐level weights were calculated based on the numbers of included and total households in each village. For binary outcomes, univariable and multivariable logistic regressions were used to obtain crude and adjusted odds ratios (OR) and 95% confidence intervals (CI). *P*‐values were calculated using Wald tests. Adjustment was made for any potential confounder for which there was evidence of crude association with the outcome or which was considered to have a possible role, *a priori*.

Raw asIgE responses were skewed. Therefore, we used simple and multiple linear regressions to examine the association between helminth infections and log_10_ levels of asIgE, and back transformed results to obtain geometric mean ratios (GMRs) and 95% CIs. For all outcomes, the role of *S. mansoni* infection intensity was assessed using the test for trend.

The population attributable fraction (PAF) for reported wheeze due to atopy was estimated as p’(OR−1)/OR with p’ the prevalence of a positive SPT response among individuals with reported wheeze and OR the odds ratio for the wheeze–SPT association. We prespecified that we would examine whether helminth infections modified the associations between the atopy and the wheeze outcomes, by fitting interaction terms in multivariable logistic regression models. We also undertook a series of additional exploratory interaction analyses between helminth infections for each outcome, in an attempt to understand the primary association findings. Finally, as most previous studies have been performed in children, we conducted an exploratory investigation into whether associations between allergy‐related outcomes and between helminths and allergy‐related outcomes differed by age group (<16 *vs* ≥16 years).

## Results

### Study participant characteristics

Of 1170 households selected, 144 (median per village 5, range 0–13) were excluded, because nobody was available to take part (*n* = 74), household members refused (*n* = 34), household was unoccupied (*n* = 28), household members’ main place of residence was another selected household (*n* = 6), and household members were ill (*n* = 2). From the remaining 1026 households, 2316 individuals were surveyed. Characteristics of the participating individuals are shown in Table 1, with further details described elsewhere 29.

**Table 1:** Characteristics of survey participants

Characteristic	*n*/*N* (%^a^)
Socio‐demographic characteristics	
Age in years, median (IQR)	24 (8, 32)
Male sex	1268/2316 (54.1)
Place of birth	
Fishing village	617/2302 (26.6)
Rural village	1381/2302 (59.2)
Town or city	304/2302 (14.1)
Occupation	
Child/student	737/2299 (32.8)
Housewife	125/2299 (6.0)
Fishing or lake related	836/2299 (36.6)
Shops, saloons, artisans, service providers	125/2299 (6.0)
Bars, restaurants, food providers, entertainment	152/2299 (5.8)
Agriculture, lumbering, charcoal	266/2299 (10.0)
Professional	15/2299 (0.9)
Unemployed	43/2299 (2.0)
Number of siblings, median (IQR)	5 (3, 8)
*P. falciparum* infection	139/2115 (7.3)
HIV infection (≥16 years)	244/1376 (17.5)
Any previous worm treatment in last 12 months	949/2284 (40.6)
Helminth infections	
*S. mansoni* (Kato‐Katz)	1041/1996 (51.4)
*S. mansoni* (urine CCA)	661/917 (72.0)
*S. mansoni* intensity (Kato‐Katz)	
Uninfected	955/1996 (48.6)
Low	429/1996 (21.0)
Moderate	288/1996 (13.7)
Heavy	324/1996 (16.6)
*N. americanus* (PCR)	453/1994 (21.9)
*N. americanus* intensity (PCR), Ct median (IQR)	35.7 (33.0–39.1)
*S. stercoralis* (PCR)	259/1994 (11.9)
*S. stercoralis* intensity (PCR), Ct median (IQR)	34.2 (31.9–7.1)
*T. trichiura* (Kato‐Katz)	230/1996 (9.8)
*T. trichiura* intensity (Kato‐Katz)	
Uninfected	1766/1996 (90.2)
Low	223/1996 (9.5)
Moderate	6/1996 (0.2)
Heavy	1/1996 (0.0004)
*M. perstans*	51/2099 (2.5)
*A. lumbricoides* (Kato‐Katz)	27/1996 (1.2)
Allergy‐related outcomes	
Wheeze in last 12 months, <5 years	12/434 (2.1)
Wheeze in last 12 months, ≥5 years	95/1862 (5.1)
Atopy (SPT)	
Any	404/1976 (19.1)
*Dermatophagoides*	190/1978 (9.0)
*Blomia*	205/1976 (9.6)
Cockroach	272.1977 (13.2)
Atopy (detectable asIgE)	
Any	1685/2116 (79.7)
*Dermatophagoides*	1534/2115 (72.7)
Cockroach	886/2117 (41.0)
Visible flexural dermatitis	15/2145 (0.7)

a Percentages adjusted for the survey design.

**Figure 1:**
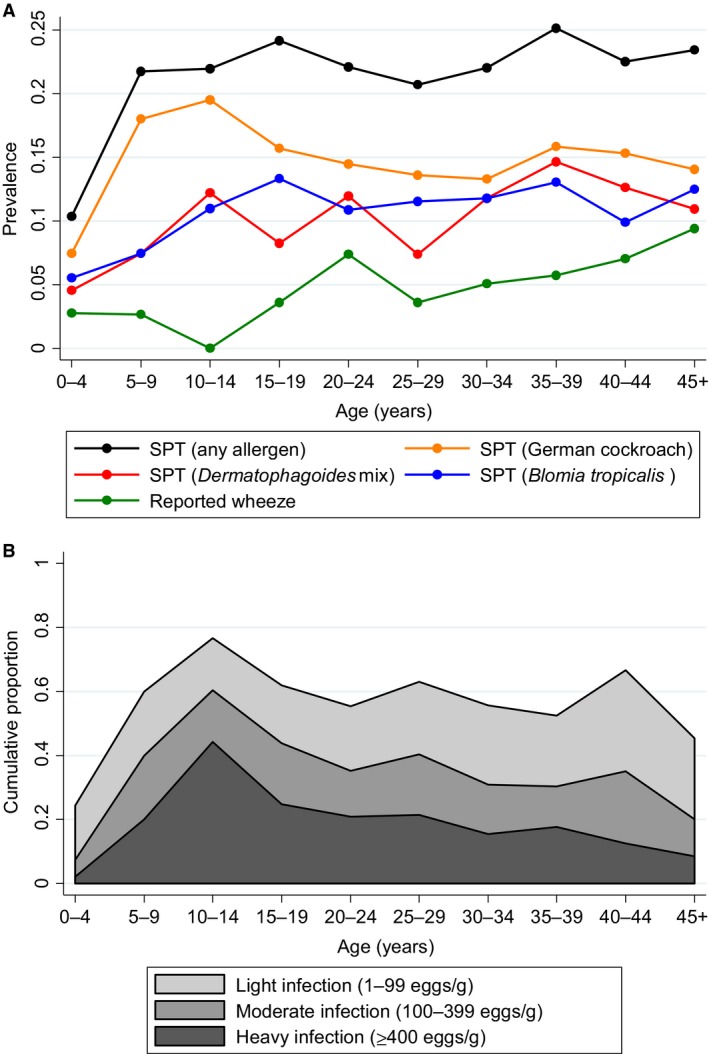
(A) Prevalence of positive SPT response (any allergen, German cockroach, *Dermatophagoides* mix, *Blomia tropicalis*) and reported wheeze in last 12 months, by age group. (B) Prevalence and intensity of *S. mansoni* infections, by age group.

Reported wheeze in the last 12 months was rare (Table 1) but increased with age (Fig. 1A); 15 participants (0.7%) had visible flexural eczema (four satisfied the UK criteria for atopic eczema). Nineteen per cent of participants were atopic based on SPT with cockroach the most common allergen to elicit a response. Prevalence of positive SPT response to cockroach peaked in school‐aged children while for other allergens, prevalence increased with age (Fig. 1A). Median (IQR) asIgE was 1440 (170–3990) ng/ml for *Dermatophagoides* and 220 (70–650) ng/ml for cockroach, with 73% of participants having detectable levels of *Dermatophagoides* IgE, 41% having detectable levels of cockroach IgE and 80% having detectable asIgE for either allergen.

The numbers and survey design‐adjusted percentages of individuals infected with each helminth are shown in Table 1. *S. mansoni* was most commonly detected, with infections peaking in prevalence and intensity among school‐aged children (Fig. 1B), followed by *N. americanus*,* S. stercoralis*,* T. trichiura*,* M. perstans* and *A. lumbricoides* (Table 1). We did not detect any *A. duodenale* among the subgroup of 200 participants investigated. A third of those infected with *S. mansoni* based on Kato‐Katz had heavy infections. For both *T. trichiura* and *A. lumbricoides,* all but seven infected individuals had light infections; therefore, we were not powered to look for associations between intensities of these helminths and the study outcomes. Infection intensities for *N. americanus* and *S. stercoralis* were generally light, with median (IQR) *C*_t_ values of 35.7 (33.0, 39.1) and 34.2 (31.9, 37.1), respectively. Based on urine CCA, 72% of 917 individuals tested were infected with *S. mansoni* (compared to 48% classified as infected by Kato‐Katz in this subgroup). Of the 421 individuals who were *S. mansoni* uninfected based on Kato‐Katz and for whom CCA results were available, 218 (52%) were positive on CCA and could be considered as having ‘very light’ infections not detected with Kato‐Katz analysis of a single stool sample.

### Associations between allergy‐related outcomes

**Figure 2:**
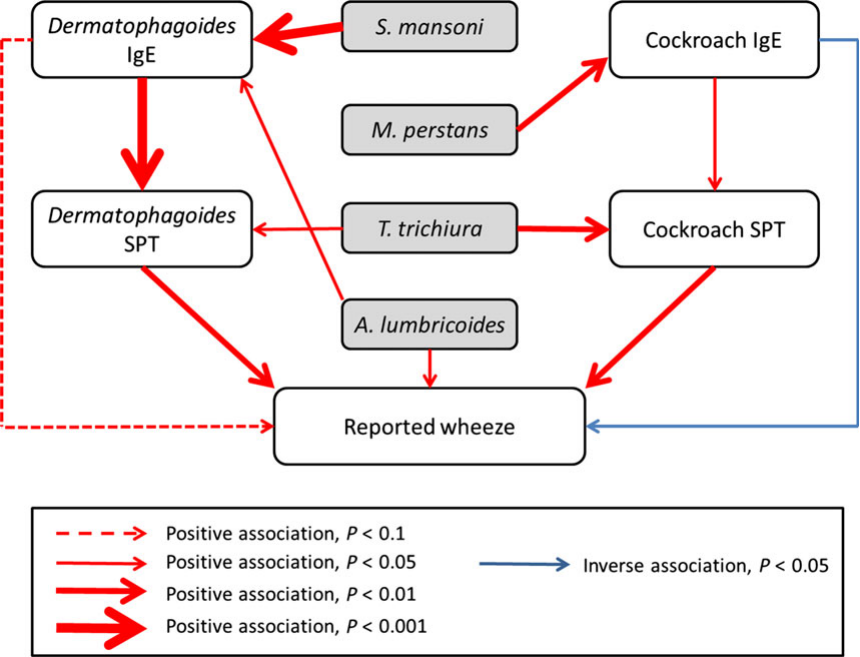
Summary of associations between helminths and allergy‐related outcomes. Red arrows denote positive associations and blue arrows denote inverse associations, with the thickness of the arrow indicating the magnitude of the *P*‐value.

Key associations between allergy‐related outcomes and between helminths and allergy‐related outcomes are summarized in Fig. 2. Individuals with a positive SPT response to any allergen were more likely to report wheeze [OR 2.49 (95% CI: 1.43, 4.33), *P* = 0.002]; the PAF for reported wheeze associated with atopy based on SPT was 19.9%. This association was seen consistently for both under‐ and over‐fives and for each of the three allergens used for SPT, and was stronger as the number of allergens for which participants had a positive SPT increased (*P*‐value for trend test <0.001). Individuals with higher *Dermatophagoides*‐specific IgE were more likely to have a positive SPT response to *Dermatophagoides* [OR for each unit increase in log *Dermatophagoides*‐specific IgE 1.69 (95% CI: 1.30, 2.20), *P* < 0.001]; cockroach‐specific IgE and cockroach‐specific SPT response were also positively associated albeit less strongly [OR 1.19 (1.04, 1.36), *P* = 0.02]. Dermatophagoides‐specific IgE level and reported wheeze (all ages) were weakly positively associated [OR 1.21 (0.96, 1.51), *P* = 0.10]; cockroach‐specific IgE level and reported wheeze were inversely associated [OR 0.77 (0.64, 0.91), *P* = 0.01].

### Associations between helminths and allergy‐related outcomes

For reported wheeze in under‐fives, we were only able to examine associations with *S. mansoni* and *T. trichiura* (as for all other helminths, no infected child had reported wheeze) and found no evidence of association with either [adjusted OR (95% CI), *P*: 2.12 (0.23, 19.20), 0.49 and 3.06 (0.65, 14.49), 0.15, respectively].

Table 2 summarizes associations between helminth infections and wheeze in over‐fives, and atopy based on SPT. Table 3 summarizes associations between helminths and asIgE response [analysed as detectable *vs* nondetectable and as log (asIgE)]. *S. mansoni* was positively associated with *Dermatophagoides*‐specific IgE [aOR for detectable *vs* nondetectable 1.43 (1.19, 1.72), *P* < 0.001 and aGMR from continuous analysis 1.64 (1.23, 2.18), *P* = 0.001, respectively]. There was a dose–response relationship, with individuals with the heaviest infections most likely to have high IgE (test for trend *P* < 0.001, Table 3). *T. trichiura* was positively associated with atopy based on SPT response [aOR 2.08 (1.38, 3.15), *P* = 0.001 for SPT to any allergen] with the strongest association seen for cockroach SPT. Individuals infected with *S. stercoralis* were somewhat more likely to have detectable cockroach‐specific IgE [aOR 1.31 (1.00, 1.72), *P* = 0.05]. Individuals with *M. perstans* were more likely to have detectable cockroach‐specific IgE and to have higher levels [aOR 2.48 (1.51, 4.07), *P* = 0.001 and aGMR 2.37 (1.39, 4.06), *P* = 0.003, respectively]. Finally, *A. lumbricoides* was positively associated with wheeze in individuals ≥5 years [aOR 6.36 (1.10, 36.63), *P* = 0.04] and with *Dermatophagoides*‐specific IgE [aOR 2.58 (1.24, 5.34), *P* = 0.01 and aGMR 2.34 (1.11, 4.95), *P* = 0.03]. No inverse associations between the helminths and the allergy‐related outcomes considered were seen.

**Table 2:** Associations between helminth infections and (i) reported wheeze in individuals aged over 5 years, (ii) positive skin prick test to *Dermatophagoides* mix, cockroach, *Blomia tropicalis*, any allergen. Adjusted associations with *P* < 0.05 are highlighted in bold

Helminth infection status	*n*/*N* (%)^a^	Adjusted OR (95% CI)^b^^,^ c	*P*
Outcome: wheeze, over 5 years			
* S. mansoni* d			
* *Uninfected	39/665 (6.5)	1	
* *Infected	49/940 (4.7)	0.81 (0.47, 1.40)	0.44
* S. mansoni* intensity^e^			
* *Uninfected	39/665 (6.5)	1	
* *Light	19/361 (4.6)	0.76 (0.42, 1.38)	0.64
* *Moderate	11/266 (4.2)	0.73 (0.32, 1.66)	(0.83)^f^
* *Heavy	19/313 (5.3)	1.00 (0.39, 2.58)	
* N. americanus*			
* *Uninfected	65/1196 (5.2)	1	
* *Infected	23/408 (6.3)	1.20 (0.60, 2.41)	0.60
* T. trichiura*			
* *Uninfected	78/1416 (5.4)	1	
* *Infected	10/189 (6.3)	1.55 (0.73, 3.30)	0.24
* S. stercoralis*			
* *Uninfected	74/1369 (5.5)	1	
* *Infected	14/235 (5.5)	0.81 (0.45, 1.47)	0.47
* M. perstans*			
* *Uninfected	90/1707 (5.3)	1	
* *Infected	4/50 (7.4)	1.27 (0.30, 5.42)	0.73
* A. lumbricoides*			
* *Uninfected	84/1585 (5.3)	**1**	
* *Infected	4/20 (19.2)	**6.36 (1.10, 36.63)**	**0.04**
Outcome: atopy (skin prick test positive to any allergen)			
* S. mansoni* d			
* *Uninfected	147/805 (16.5)	1	
* *Infected	215/965 (20.6)	1.13 (0.86, 1.47)	0.37
* S. mansoni* intensity^e^			
* *Uninfected	147/805 (16.5)	1	
* *Light	96/395 (22.5)	1.26 (0.85, 1.86)	0.67
* *Moderate	56/271 (19.6)	1.02 (0.74, 1.41)	(0.96)^f^
* *Heavy	63/299 (19.2)	1.02 (0.70, 1.50)	
* N. americanus*			
* *Uninfected	290/1367 (19.5)	1	
* *Infected	71/400 (15.9)	0.72 (0.47, 1.10)	0.12
* T. trichiura*			
* *Uninfected	301/1567 (17.6)	**1**	
* *Infected	61/203 (29.7)	**2.08 (1.38, 3.15)**	**0.001**
* S. stercoralis*			
* *Uninfected	327/1532 (19.6)	1	
* *Infected	34/235 (12.5)	0.55 (0.29, 1.06)	0.07
* M. perstans*			
* *Uninfected	392/1900 (19.4)	1	
* *Infected	9/48 (16.0)	0.68 (0.28, 1.68)	0.39
* A. lumbricoides*			
* *Uninfected	356/1744 (18.7)	1	
* *Infected	6/26 (23.4)	1.31 (0.40, 4.26)	0.64
Outcome: atopy (skin prick test positive to *Dermatophagoides*)			
* S. mansoni* d			
* *Uninfected	68/806 (7.6)	1	
* *Infected	104/966 (9.9)	1.13 (0.89, 1.44)	0.31
*S. mansoni* intensity^e^			
Uninfected	68/806 (7.6)	1	
Light	46/395 (10.0)	1.12 (0.74, 1.70)	0.74
Moderate	28/271 (10.1)	1.17 (0.76, 1.80)	(0.50)^f^
Heavy	30/300 (9.6)	1.11 (0.74, 1.65)	
*N. americanus*			
Uninfected	139/1369 (9.3)	1	
Infected	32/400 (7.3)	0.72 (0.43, 1.21)	0.20
*T. trichiura*			
Uninfected	147/1569 (8.4)	**1**	
Infected	25/203 (12.7)	**1.73 (1.03, 2.90)**	**0.04**
*S. stercoralis*			
Uninfected	152/1534 (8.9)	1	
Infected	19/235 (8.0)	0.84 (0.38, 1.86)	0.65
*M. perstans*			
Uninfected	184/1902 (9.1)	1	
Infected	3/48 (7.1)	0.78 (0.27, 2.26)	0.63
*A. lumbricoides*			
Uninfected	168/1746 (8.7)	1	
Infected	4/26 (17.6)	2.46 (0.73, 8.31)	0.14
Outcome: atopy (skin prick test positive to cockroach)			
*S. mansoni* d			
Uninfected	92/805 (10.5)	1	
Infected	152/966 (14.8)	1.29 (0.88, 1.90)	0.18
*S. mansoni* intensity^e^			
Uninfected	92/805 (10.5)	1	
Light	69/395 (17.1)	1.58 (0.96, 2.61)	0.20
Moderate	41/271 (13.8)	1.14 (0.75, 1.72)	(0.95)^f^
Heavy	42/300 (12.8)	1.02 (0.60, 1.73)	
*N. americanus*			
Uninfected	19/1368 (13.1)	1	
Infected	51/400 (11.7)	0.73 (0.40, 1.31)	0.27
*T. trichiura*			
Uninfected	198/1568 (11.8)	**1**	
Infected	46/203 (21.8)	**1.98 (1.30, 3.01)**	**0.003**
*S. stercoralis*			
Uninfected	219/1533 (13.3)	1	
Infected	25/235 (9.3)	0.62 (0.32, 1.18)	0.14
*M. perstans*			
Uninfected	267/1901 (13.5)	1	
Infected	5/48 (9.4)	0.58 (0.21, 1.62)	0.28
*A. lumbricoides*			
Uninfected	240/1745 (12.8)	1	
Infected	4/26 (15.0)	1.06 (0.33, 3.37)	0.92
Outcome: atopy (skin prick test positive to *Blomia tropicalis*)			
*S. mansoni* d			
Uninfected	81/805 (8.5)	1	
Infected	98/965 (9.7)	1.02 (0.66, 1.59)	0.92
*S. mansoni* intensity^e^			
Uninfected	81/805 (8.5)	1	
Light	42/395 (9.2)	0.97 (0.58, 1.63)	0.89
Moderate	23/271 (8.6)	0.95 (0.59, 1.52)	(0.65)^f^
Heavy	33/299 (11.2)	1.18 (0.64, 2.19)	
*N. americanus*			
Uninfected	139/1367 (9.2)	1	
Infected	39/400 (8.8)	0.89 (0.56, 1.44)	0.63
*T. trichiura*			
Uninfected	155/1567 (8.8)	1	
Infected	24/203 (11.8)	1.45 (0.79, 2.56)	0.22
*S. stercoralis*			
Uninfected	163/1532 (9.5)	1	
Infected	15/235 (6.3)	0.59 (0.26, 1.34)	0.20
*M. perstans*			
Uninfected	195/1900 (9.6)	1	
Infected	7/48 (13.8)	1.37 (0.58, 3.26)	0.45
*A. lumbricoides*			
Uninfected	174/1744 (9.0)	1	
Infected	5/26 (21.7)	3.73 (0.91, 15.26)	0.07

a Percentages adjusted for the survey design.

b Adjusted odds ratio (OR) and 95% confidence intervals (CI) adjusted for survey design.

c All adjusted ORs adjusted for age, sex, occupation, number of siblings. For associations with wheeze, all ORs were also adjusted for household asset score and household crowding and helminth‐specific ORs were additionally adjusted as follows: *S. mansoni* – malaria infection, hand‐washing behaviour, *A. lumbricoides* infection; hookworm – malaria infection, hand‐washing behaviour, HIV infection, *A. lumbricoides* infection; *T. trichiura* – hand‐washing behaviour; *M. perstans* – malaria infection; *A. lumbricoides* – maternal history of asthma. For associations with SPT (any and allergen‐specific), all ORs were also adjusted for number of siblings, area of birth, and helminth‐specific ORs were additionally adjusted as follows: *S. mansoni* – *T. trichiura* infection; hookworm – *S. mansoni* infection, *S. stercoralis* infection, *T. trichiura* infection. For associations with *Dermatophagoides* SPT, helminth‐specific ORs were additionally adjusted as follows*: S. mansoni* – maternal tribe, hand‐washing behaviour; hookworm – household toilet access; *T. trichiura* – maternal tribe, household toilet access; *M. perstans* – maternal tribe. For associations with cockroach SPT, helminth‐specific ORs were additionally adjusted as follows: hookworm – household asset score; *T. trichiura* – household toilet access; *S. stercoralis* – household asset score. For associations with *Blomia tropicalis* SPT, ORs for *S. mansoni* and hookworm were additionally adjusted for hand‐washing behaviour.

d Based on Kato‐Katz.

e Light: 1–99 eggs per gram, moderate: 100–399 eggs per gram, heavy: ≥400 eggs per gram.

f *P*‐value for test for trend.

**Table 3:** Associations between helminth infections and allergen‐specific IgE. Adjusted associations with *P* < 0.05 are highlighted in bold

Helminth infection status	*n*/*N* (%)/geometric mean^a^	Adjusted OR/GMR (95% CI)^b^^,^ c	*P*
Outcome: atopy (Detectable *Dermatophagoides*‐specific IgE)			
*S. mansoni* d			
Uninfected	588/873 (67.4)	**1**	
Infected	771/1007 (76.8)	**1.43 (1.19, 1.72)**	**<0.001**
*S. mansoni* intensity^e^			
Uninfected	588/873 (67.4)	**1**	
Light	298/411 (71.8)	**1.13 (0.89, 1.42)**	**0.002**
Moderate	216/279 (79.3)	**1.69 (1.22, 2.35)**	**(<0.001)** f
Heavy	257/317 (81.0)	**1.95 (1.28, 2.95)**	
*N. americanus*			
Uninfected	1029/1446 (71.4)	1	
Infected	329/432 (75.9)	1.10 (0.75, 1.61)	0.60
*T. trichiura*			
Uninfected	1188/1659 (71.9)	1	
Infected	171/221 (76.7)	1.13 (0.82, 1.56)	0.44
*S. stercoralis*			
Uninfected	1169/1630 (71.5)	1	
Infected	189/248 (79.0)	1.26 (0.95, 1.67)	0.11
*M. perstans*			
Uninfected	1483/2044 (72.7)	1	
Infected	40/51 (83.8)	1.53 (0.68, 3.41)	0.29
*A. lumbricoides*			
Uninfected	1336/1853 (72.2)	**1**	
Infected	23/27 (88.0)	**2.58 (1.24, 5.34)**	**0.01**
Outcome: atopy (Detectable cockroach‐specific IgE)			
*S. mansoni* d			
Uninfected	362/874 (41.0)	1	
Infected	425/1008 (41.6)	0.88 (0.58, 1.35)	0.56
*S. mansoni* intensity^e^			
Uninfected	362/874 (41.0)	1	
Light	174/411 (40.4)	0.85 (0.52, 1.39)	0.92
Moderate	112/279 (43.3)	0.94 (0.48, 1.82)	(0.62)^f^
Heavy	139/318 (41.7)	0.89 (0.57, 1.39)	
*N. americanus*			
Uninfected	598/1447 (40.4)	1	
Infected	188/433 (44.2)	1.06 (0.75, 1.49)	0.73
*T. trichiura*			
Uninfected	690/1660 (41.1)	1	
Infected	97/222 (43.1)	1.03 (0.85, 1.24)	0.76
*S. stercoralis*			
Uninfected	667/1632 (40.4)	1	
Infected	119/248 (47.3)	1.31 (1.00, 1.72)	0.05
*M. perstans*			
Uninfected	852/2046 (40.7)	**1**	
Infected	31/51 (61.9)	**2.48 (1.51, 4.07)**	**0.001**
*A. lumbricoides*			
Uninfected	776/1855 (41.3)	1	
Infected	11/27 (42.6)	0.96 (0.55, 1.67)	0.87
Outcome: level of Dermatophagoides‐specific IgE			
*S. mansoni* d			
Uninfected	253	**1**	
Infected	530	**1.64 (1.23, 2.18)**	**0.001**
*S. mansoni* intensity^e^			
Uninfected	253	**1**	
Light	344	**1.14 (0.77, 1.69)**	**0.001**
Moderate	602	**1.94 (1.31, 2.87)**	**(<0.001)** f
Heavy	817	**2.69 (1.46, 4.95)**	
*N. americanus*			
Uninfected	350	1	
Infected	475	1.09 (0.65, 1.83)	0.72
*T. trichiura*			
Uninfected	361	1	
Infected	523	1.24 (0.78, 1.99)	0.35
*S. stercoralis*			
Uninfected	350	1	
Infected	613	1.27 (0.92, 1.77)	0.14
*M. perstans*			
Uninfected	378	1	
Infected	997	1.69 (0.73, 3.90)	0.21
*A. lumbricoides*			
Uninfected	370	**1**	
Infected	970	**2.34 (1.11, 4.95)**	**0.03**
Outcome: level of cockroach‐specific IgE			
*S. mansoni* d			
Uninfected	164	1	
Infected	165	0.78 (0.54, 1.13)	0.18
*S. mansoni* intensity^e^			
Uninfected	164	1	
Light	158	0.76 (0.51, 1.21)	0.53
Moderate	174	0.77 (0.37, 1.61)	(0.26)^f^
Heavy	167	0.84 (0.57, 1.22)	
*N. americanus*			
Uninfected	155	1	
Infected	201	1.12 (0.84, 1.48)	0.42
*T. trichiura*			
Uninfected	162	1	
Infected	194	1.17 (0.93, 1.47)	0.17
*S. stercoralis*			
Uninfected	162	1	
Infected	180	0.98 (0.62, 1.54)	0.92
*M. perstans*			
Uninfected	162	**1**	
Infected	428	**2.37 (1.39, 4.06)**	**0.003**
*A. lumbricoides*			
Uninfected	164	1	
Infected	242	1.57 (0.85, 2.89)	0.15

a For binary outcomes, figures shown are *n*/*N* with percentages adjusted for the survey design. For continuous outcomes, figures shown are geometric means adjusted for the survey design.

b For binary outcomes, figures shown are adjusted odds ratios (OR) [95% confidence intervals (CI)] adjusted for survey design. For continuous outcomes, figures shown are adjusted geometric mean ratios (GMR) (95% CI) adjusted for survey design.

c All adjusted ORs adjusted for age, sex, occupation, area of birth. For associations with detectable *Dermatophagoides* IgE, ORs were additionally adjusted as follows: *S. mansoni* – preschool attendance, *A. lumbricoides* infection; hookworm – *S. mansoni* infection, *S. stercoralis* infection; *T. trichiura* – household crowding, animal ownership, *S. mansoni* infection, *A. lumbricoides* infection; *S. stercoralis* – household crowding, animal ownership; *A. lumbricoides* – *S. mansoni* infection. For associations with detectable cockroach IgE, ORs were additionally adjusted as follows: hookworm –*S. stercoralis* infection; *T. trichiura* – animal ownership; *S. stercoralis* – animal ownership.

All adjusted GMRs adjusted for age, sex, occupation, area of birth. For associations with *Dermatophagoides* IgE, GMRs were additionally adjusted as follows: *S. mansoni* – number of siblings, preschool attendance, hand‐washing behaviour, *A. lumbricoides* infection; hookworm – number of siblings, malaria infection, *S. mansoni* infection, *S. stercoralis* infection; *T. trichiura* – household crowding, hand‐washing behaviour, animal ownership, *S. mansoni* infection, *A. lumbricoides* infection; *S. stercoralis* – number of siblings, household crowding and animal ownership; *M. perstans* – malaria infection; *A. lumbricoides* – *S. mansoni* infection. For associations with cockroach IgE, GMRs were additionally adjusted as follows: *S. mansoni* – maternal history of asthma, hookworm infection; hookworm – anthelminthic treatment in last year; *T. trichiura* – HIV infection, animal ownership, hookworm infection; *S. stercoralis* – HIV infection, animal ownership, anthelminthic treatment in last year.

d Based on Kato‐Katz.

e Light: 1–99 eggs per gram, moderate: 100–399 eggs per gram, heavy: ≥400 eggs per gram.

f *P*‐value for test for trend.

### Investigation of effect modification by helminths and age group

The positive association between *Dermatophagoides* IgE level and wheeze was only seen among those infected with *S. mansoni* [OR = 1.75, (1.22, 2.52), *P* = 0.004] and not in uninfected individuals [OR = 1.04 (0.81, 1.33), *P* = 0.77; interaction *P* = 0.01], Table S1. A similar pattern was seen for the association between cockroach SPT and wheeze [OR = 3.27 (2.08, 5.14), *P* < 0.001 among *S. mansoni*‐infected individuals, OR = 1.37 (0.52, 3.61), *P* = 0.51 among *S. mansoni*‐uninfected individuals, interaction *P* = 0.09, Table S1]. Conversely, there was some evidence that the positive association between cockroach‐specific IgE and SPT was suppressed among those infected with hookworm [OR = 0.95 (0.72, 1.26), *P* = 0.72 among hookworm‐infected individuals, OR = 1.38 (1.10, 1.74), *P* = 0.008 among hookworm‐uninfected individuals, interaction *P* = 0.11, Table S1]. No interactive effects of other helminths were seen. The inverse association between cockroach IgE and reported wheeze was only seen among adults [OR = 0.70 (0.59, 0.83), *P* < 0.001 among those aged ≥16 years), OR = 1.40 (0.98, 2.02), *P* = 0.07 among those aged <16 years, interaction *P* = 0.002].

The positive associations between *T. trichiura* and SPT response and between *A. lumbricoides* and wheeze in over‐fives were enhanced by concurrent infection with *S. stercoralis* (interaction *P* = 0.004 and *P* < 0.001, respectively; Table 4) and the former was also enhanced by concurrent hookworm (interaction *P* = 0.05; Table 4). *M. perstans* infection was associated with higher levels of *Dermatophagoides*‐specific IgE in the absence of concurrent *S. mansoni* infection (interaction *P* = 0.007). There was no evidence of effect modification between age group and helminths for any outcome.

**Table 4:** Interactions between helminth infections in their association with allergy‐related outcomes

Helminth infection status					
*S. stercoralis* status	*T. trichiura* status	*n*/*N* (%)^a^	Adjusted OR (95% CI)^b^^,^ c	*P*	Interaction *P*‐value
Outcome: SPT response for any allergen					
* *Uninfected	Uninfected	279/1364 (18.7)	1		0.004
	Infected	48/168 (28.0)	1.85 (1.18, 2.88)	0.01	
Infected	Uninfected	21/201 (8.9)	1		
	Infected	13/34 (40.2)	9.88 (2.27, 43.01)	0.004	
* N. americanus* status	*T. trichiura* status				
Uninfected	Uninfected	251/1234 (18.7)	1		0.05
	Infected	39/133 (28.4)	1.73 (1.10, 2.73)	0.02	
Infected	Uninfected	49/331 (13.0)	1		
	Infected	22/69 (32.6)	4.06 (2.09, 7.88)	<0.001	
*S. stercoralis* status	*A. lumbricoides* status	*n*/*N* (%)^a^	Adjusted OR (95% CI)^b^^,^ c	*P*	Interaction *P*‐value
Outcome: wheeze, over 5 years					
* *Uninfected	Uninfected	73/1352 (5.5)	1		<0.001
	Infected	1/16 (7.7)	1.63 (0.13, 19.80)	0.69	
Infected	Uninfected	11/231 (4.5)	1		
	Infected	3/4 (71.6)	485.9 (5.71, 41 341.5)	0.008	
*S. mansoni* status	*M. perstans* status	Geometric mean (ng/ml)^a^	Crude GMR (95% CI)^b^^,^ c	*P*	Interaction *P*‐value
Outcome: *Dermatophagoides*‐specific IgE					
Uninfected	Uninfected (*n* = 839)	27.9	1		0.007
	Infected (*n* = 19)	113.7	3.12 (1.67, 5.80)	0.001	
Infected	Uninfected (*n* = 979)	47.6	1		
	Infected (*n* = 25)	57.2	0.94 (0.50, 1.78)	0.85	

a For binary outcomes, figures shown are *n*/*N* with percentages adjusted for the survey design. For continuous outcome, figures shown are geometric means adjusted for the survey design.

b For binary outcomes, figures shown are adjusted odds ratios (OR) [95% confidence intervals (CI)] allowing for survey design. For continuous outcome, figures shown are adjusted geometric mean ratios (GMR) (95% CI) allowing for survey design.

c All adjusted OR/GMR adjusted for age, sex, number of siblings, occupation. Associations for SPT additionally adjusted for area of birth, associations for wheeze additionally adjusted for household crowding and asset score, associations for IgE additionally adjusted for area of birth, preschool attendance, malaria infection, hand‐washing behaviour.

## Discussion

In these remote fishing communities of Lake Victoria where helminths are highly prevalent, atopy was more common in individuals infected with *Trichuris*, schistosomiasis*, M. perstans* or *Ascaris*, and reported wheeze in the last year was more common among those with *Ascaris* infection. Our findings are in contrast to the hypothesis that chronic helminth infections protect against atopy and allergy‐related diseases.

We explored various explanations for why our findings might differ from those reported by others. Concurrent infection with other helminths or pathogens could be a factor: we found some evidence that the association between *Trichuris* infection and SPT response was enhanced by concurrent infection with *S. stercoralis* or hookworm (Table 4); these results could also be interpreted as indicating that *S. stercoralis* and hookworm infections were inversely associated with atopy, in the absence of *Trichuris* co‐infection. The fact that our survey was not restricted to children (unlike most other studies) is unlikely to be the explanation: although we found that the inverse association between cockroach IgE and reported wheeze was not seen in children, there was otherwise no evidence for effect modification by age. Our findings were consistent for both cockroach and dust mite allergens; thus, differences between studies in allergens used is unlikely to explain our different findings. Another possible explanation that we cannot exclude is that individuals in our study setting may have suffered more long‐term and chronic infections compared to those in other studies. Finally, although hookworm infection as detected by PCR was fairly common, it was generally of low intensity; hence, this may have reduced our ability to detect associations for this helminth.

We estimate that in this setting, the population fraction of reported wheeze attributable to atopy is around 20%, lower than reported in most high‐income settings, but similar to many other LICs 9, 38. Consistent with findings from other developing country settings 21, hookworm infection appeared to suppress associations between IgE and SPT for cockroach responses; however, schistosomiasis had the opposite effect, promoting associations between atopy and wheeze for both dust mite and cockroach allergens.

Helminth infections could promote atopy either by nonspecifically driving the antigen presentation‐T‐cell‐to‐B‐cell immune response axis towards greater production of IgE, or by inducing cross‐reactive IgE. Regarding the latter, asthma severity has been shown to be related to *Ascaris* IgE levels, which correlate with mite‐specific IgE 39, cross‐reactivity has been demonstrated for selected *Ascaris* and mite antigens 40, and immunization of rabbits with *Ascaris* antigens induces IgE which cross‐reacts with house dust mite 41. This accords with our observed association between *Ascaris* and elevated dust mite‐specific IgE. Likewise, molecular modelling indicates that *S. mansoni* contains cysteine proteases homologous to the house dust mite antigen Der p 1 42 and *S. mansoni* infection was associated with elevated house dust mite‐specific IgE in our study. Cross‐reactive IgE can also occur to molecules such as tropomyosin which are highly conserved between invertebrates (including nematodes, schistosomes, mites and cockroach) 43 and, consistent with cross‐reactivity, the prevalence of positive SPT responses to cockroach and the heaviest *S. mansoni* infections both peaked in school‐aged children in our study.

Mechanisms for a positive association between helminths and wheeze could include exacerbation of allergen‐specific atopic responses or a direct response to the allergen‐like helminth proteins experienced during larval migration through the lungs. The former might explain our finding that concurrent *S. mansoni* infection strengthens the association between allergen‐specific IgE and wheeze. The latter may explain the association between *Ascaris* and wheeze – the long‐recognized Löffler's syndrome 44 – although cross‐reactivity between Ascaris and mite allergens may also contribute, as discussed above.

For each of these possible mechanisms, concurrent infection with a helminth species that down‐modulates allergy‐related immune responses might modify the association between pro‐allergenic helminth species and allergy‐related outcomes in the same way that hookworm has been observed to modify the link between allergen‐specific IgE and histamine release 21. However, our interaction analyses showed little evidence of such effects: the only result consistent with this hypothesis was that *S. mansoni* infection modified the association between *M. perstans* and house dust mite‐specific IgE production.

Despite the positive associations observed between helminths and allergy‐related outcomes in this study, the overall prevalence of wheeze, eczema and SPT positivity was low compared with developed countries and urban settings in low or middle‐income countries 8, 45. This suggests that factors other than current active helminth infection in these communities have important protective effects against allergy‐related outcomes. These factors could include prenatal exposure to helminths 22, 46, exposures to a myriad of infectious, xenobiotic or commensal organisms 47, 48, and a range of life‐style factors 49, which await further investigation in this setting.

We found high levels of allergen‐specific IgE. This could be a consequence of immunological cross‐reactivity between helminth allergens and aeroallergens or a result of nonspecific stimulation of IgE production resulting from intense helminth exposure (as discussed above), or an artefact of our in‐house assay. Further studies to determine the characteristics of the IgE present in this population are in progress.

Our study had some limitations. We could only evaluate helminth infections endemic to our study setting: for example, for hookworm, we could investigate associations between *N. americanus* and allergy outcomes, but not *A. duodenale*, as the latter was not found. The cross‐sectional design of this survey means that, strictly speaking, we cannot tell the relative timing of allergen sensitization and helminth exposure. However, our age‐prevalence profiles show that both prenatal helminth exposure and infection in infancy and early childhood are likely in this setting so prior exposure to helminths, or concurrent exposure to helminths and allergens, is likely to have occurred. Although the use of reported wheeze in the last 12 months has been validated as a proxy measure for asthma in many settings 50, there is no direct translation of the word ‘wheeze’ in the local language; thus, this outcome is likely to be subject to misclassification. Indeed, the increasing prevalence of reported wheeze with age that we observed could indicate that the phenotype being captured was to some extent related to chronic bronchitis rather than asthma. We investigated the use of a video questionnaire for wheeze in the study and found that agreement between the two approaches was fairly low although participants reporting wheeze had, on average, reduced levels of lung function parameters 29. Although we collected data on reported allergic rhinitis (a common disease caused by the allergens tested in our study, in some settings) in our survey and investigated it as an exploratory outcome, we found it to be uncommon and there was no evidence of association with any helminth; however, it is possible that this outcome was subject to misclassification, for similar reasons to wheeze. An additional source of misclassification is that we used single stool samples to assess helminth infection status 51, 52. Indeed for the subgroup of participants who underwent urine CCA testing, the prevalence of *S. manson*i was found to be much higher than when tested using Kato‐Katz of the single stool sample. Also, the PCR method used had limited sensitivity for *Strongyloides. *This could have led to underestimation of the size of any true association. The study involved a large number of statistical tests, for which we made no formal adjustment; however, the consistent patterns of positive associations are unlikely to be explained by chance. Findings from our interaction analyses should be treated with caution. Although not all of these analyses were preplanned, we felt they were important to try to shed light on our unexpected findings.

In conclusion, we found that certain helminth infections were positively associated with allergy‐related outcomes in this setting, with inverse associations only being seen in subgroup analyses. The LaVIISWA trial is currently ongoing and the impact of intensive *vs* standard anthelminthic treatment will be investigated in a further cross‐sectional household survey in 2016. At that time, we will be able to assess not only the direct impact of worm removal on allergy‐related outcomes, but also the effect of the trial interventions on the associations reported herein. If there is a causal relationship underlying the observed associations, then the allergy‐related outcome prevalence might reduce with the removal of helminth infection, rather than increasing as initially hypothesized.

## LaVIISWA trial team

Project leaders: Margaret Nampijja, Richard Sanya, Barbara Nerima. Statisticians and data managers: Emily Webb, Lawrence Muhangi, Beatrice Mirembe, Justin Okello, Jonathan Levin. Clinical officer: Milly Namutebi, Christopher Zziwa. Nurses: Esther Nakazibwe, Josephine Tumusiime. Internal monitor: Mirriam Akello. Field workers: Robert Kizindo, Moses Sewankambo, Denis Nsubuga. Laboratory staff: Stephen Cose, Linda Wammes, Proscovia Kabubi, Emmanuel Niwagaba, Gloria Oduru, Grace Kabami, Elson Abayo, Joyce Kabagenyi, Gyaviira Nkurunungi, Fred Muwonge, Dennison Kizito. Boatman: David Abiriga. HIV counselling and testing: Victoria Nannozi. Vector Control Programme staff: James Kaweesa, Edridah Tukahebwa. Principal investigator: Alison Elliott.

## Conflict of interest

The authors declare that they have no conflicts of interest.

## Author contributions

AME conceived the study. AME, ELW, ET and M Nampijja participated in designing the parent trial. AME, M Nampijja, J Kaweesa, RK, EN and M Namutebi led and participated in the survey. GO and PK ran the field laboratory, while J Kabagenyi, GN, DK and BN participated in establishing and conducting immunological assays and PCR. JJV trained and assisted in stool PCR. MA monitored the study activities. LM managed the database. ELW conducted the statistical analysis. ELW, M Nampijja and AME drafted the manuscript and all authors reviewed and contributed to it. All authors read and approved the final version of the manuscript.

## Supporting information


**Table S1.** Associations between allergy outcomes stratified by *S. mansoni* and *N. americanus* status.
