# Supplementary material for: Helminths are positively associated with atopy and wheeze in Ugandan fishing communities: results from a cross‐sectional survey
Source: Allergy. 2016 May 20;71(8):1156–69. doi: 10.1111/all.12867 (PMC4949563; doi:10.1111/all.12867)
Supplement: Supplementary file 1 — Table S1. Associations between allergy outcomes stratified by S. mansoni and N. americanus status. [file ALL-71-1156-s001.docx]

**Supplementary Table 1. Associations between allergy outcomes stratified by *S. mansoni* and *N. americanus* status.**

| ***S. mansoni*** | | | | ***N. americanus*** | | | |
| --- | --- | --- | --- | --- | --- | --- | --- |
| **Infection status** | **OR (95% CI)** | **p** | **Interaction p-value** | **Infection status** | **OR (95% CI)** | **p** | **Interaction p-value** |
| **Outcomes: *Dermatophagoides* SPT and reported wheeze** | | | | | | | |
| Uninfected | 2.18 (0.64, 7.40) | 0.20 | 0.53 | Uninfected | 2.59 (1.15, 5.84) | 0.02 | 0.71 |
| Infected | 3.32 (1.85, 5.97) | <0.001 |  | Infected | 3.37 (1.23, 9.21) | 0.02 |  |
| **Outcomes: *Dermatophagoides* IgE level and *Dermatophagoides* SPT** | | | | | | | |
| Uninfected | 1.55 (1.03, 2.35) | 0.04 | 0.37 | Uninfected | 1.68 (1.21, 2.34) | 0.003 | 0.54 |
| Infected | 1.88 (1.41, 2.51) | <0.001 |  | Infected | 1.95 (1,41, 2.69) | <0.001 |  |
| **Outcomes: *Dermatophagoides* IgE level and reported wheeze** | | | | | | | |
| Uninfected | 1.04 (0.81, 1.33) | 0.77 | 0.01 | Uninfected | 1.32 (0.99, 1.77) | 0.06 | 0.29 |
| Infected | 1.75 (1.22, 2.52) | 0.004 |  | Infected | 1.01 (0.64, 1.59) | 0.96 |  |
| **Outcomes: Cockroach SPT and reported wheeze** | | | | | | | |
| Uninfected | 1.37 (0.52, 3.61) | 0.51 | 0.09 | Uninfected | 1.91 (1.05, 3.49) | 0.04 | 0.19 |
| Infected | 3.27 (2.08, 5.14) | <0.001 |  | Infected | 3.59 (1.84, 7.02) | 0.001 |  |
| **Outcomes: Cockroach IgE level and cockroach SPT** | | | | | | | |
| Uninfected | 1.15 (0.86, 1.55) | 0.34 | 0.47 | Uninfected | 1.38 (1.10, 1.74) | 0.008 | 0.11 |
| Infected | 1.35 (1.06, 1.73) | 0.02 |  | Infected | 0.95 (0.72, 1.26) | 0.72 |  |
| **Outcomes: Cockroach IgE level and reported wheeze** | | | | | | | |
| Uninfected | 0.75 (0.55, 1.02) | 0.06 | 0.96 | Uninfected | 0.80 (0.60, 1.07) | 0.12 | 0.39 |
| Infected | 0.75 (0.60, 0.95) | 0.02 |  | Infected | 0.62 (0.41, 0.93) | 0.02 |  |
